# TBCRC 022: A Phase II Trial of Neratinib and Capecitabine for Patients With Human Epidermal Growth Factor Receptor 2–Positive Breast Cancer and Brain Metastases

**DOI:** 10.1200/JCO.18.01511

**Published:** 2019-03-12

**Authors:** Rachel A. Freedman, Rebecca S. Gelman, Carey K. Anders, Michelle E. Melisko, Heather A. Parsons, Anne M. Cropp, Kelly Silvestri, Christine M. Cotter, Kathryn P. Componeschi, Juan M. Marte, Roisin M. Connolly, Beverly Moy, Catherine H. Van Poznak, Kimberly L. Blackwell, Shannon L. Puhalla, Rachel C. Jankowitz, Karen L. Smith, Nuhad Ibrahim, Timothy J. Moynihan, Ciara C. O’Sullivan, Julie Nangia, Polly Niravath, Nadine Tung, Paula R. Pohlmann, Robyn Burns, Mothaffar F. Rimawi, Ian E. Krop, Antonio C. Wolff, Eric P. Winer, Nancy U. Lin, on behalf of the Translational Breast Cancer Research Consortium

**Affiliations:** ^1^ Dana-Farber Cancer Institute, Boston, MA; ^2^ University of North Carolina at Chapel Hill, Chapel Hill, NC; ^3^ University of California at San Francisco, San Francisco, CA; ^4^ Johns Hopkins School of Medicine, Baltimore, MD; ^5^ Massachusetts General Hospital, Boston, MA; ^6^ University of Michigan, Ann Arbor, MI; ^7^ Duke University Medical Center, Durham, NC; ^8^ University of Pittsburgh Cancer Institute, Pittsburgh, PA; ^9^ The University of Texas MD Anderson Cancer Center, Houston, TX; ^10^ Mayo Clinic, Rochester, MN; ^11^ Baylor College of Medicine, Houston, TX; ^12^ Beth Israel Deaconess Medical Center, Boston, MA; ^13^ Lombardi Comprehensive Cancer Center, Washington, DC; ^14^ The Emmes Corporation, Rockville, MD

## Abstract

**PURPOSE:**

Evidence-based treatments for metastatic, human epidermal growth factor receptor 2 (HER2)–positive breast cancer to the CNS are limited. We previously reported modest activity of neratinib monotherapy for HER2-positive breast cancer brain metastases. Here we report the results from additional study cohorts.

**PATIENTS AND METHODS:**

Patients with measurable, progressive, HER2-positive brain metastases (92% after receiving CNS surgery and/or radiotherapy) received neratinib 240 mg orally once per day plus capecitabine 750 mg/m^2^ twice per day for 14 days, then 7 days off. Lapatinib-naïve (cohort 3A) and lapatinib-treated (cohort 3B) patients were enrolled. If nine or more of 35 (cohort 3A) or three or more of 25 (cohort 3B) had CNS objective response rates (ORR), the drug combination would be deemed promising. The primary end point was composite CNS ORR in each cohort separately, requiring a reduction of 50% or more in the sum of target CNS lesion volumes without progression of nontarget lesions, new lesions, escalating steroids, progressive neurologic signs or symptoms, or non-CNS progression.

**RESULTS:**

Forty-nine patients enrolled in cohorts 3A (n = 37) and 3B (n = 12; cohort closed for slow accrual). In cohort 3A, the composite CNS ORR = 49% (95% CI, 32% to 66%), and the CNS ORR in cohort 3B = 33% (95% CI, 10% to 65%). Median progression-free survival was 5.5 and 3.1 months in cohorts 3A and 3B, respectively; median survival was 13.3 and 15.1 months. Diarrhea was the most common grade 3 toxicity (29% in cohorts 3A and 3B).

**CONCLUSION:**

Neratinib plus capecitabine is active against refractory, HER2-positive breast cancer brain metastases, adding additional evidence that the efficacy of HER2-directed therapy in the brain is enhanced by chemotherapy. For optimal tolerance, efforts to minimize diarrhea are warranted.

## INTRODUCTION

Approximately one half of patients with metastatic, human epidermal growth factor receptor 2 (HER2)–positive breast cancer will develop brain metastases,^1-8^ a clinical situation for which treatments are limited; there are no US Food and Drug Administration–approved brain-metastasis–specific systemic treatments. Although the survival of women with HER2-positive breast cancer brain metastases has improved over time,^7-10^ recurrent CNS events remain a major source of morbidity and mortality for a substantial proportion of patients.

**FIG A1.**
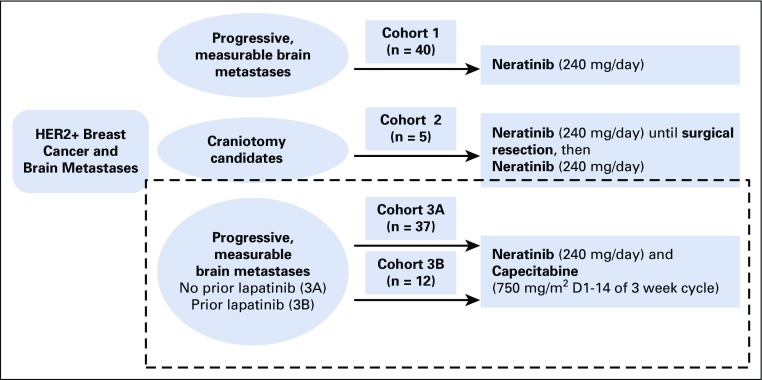
Translational Breast Cancer Research Consortium 022 study cohorts to date. Cohorts 3A and 3B are presented here. HER2, human epidermal growth factor receptor 2.

We previously reported results from cohort 1 of our multicenter phase II study^11^ (Translational Breast Cancer Research Consortium [TBCRC] 022), which evaluated the efficacy of neratinib, an irreversible pan-HER tyrosine kinase inhibitor that inhibits signal transduction through erbB1, HER2, and erbB4,^12,13^ in 40 patients with progressive HER2-positive brain metastases (CNS response = 8%).^11^ Cohort 2 was an exploratory cohort evaluating the effects of neratinib administered as a brief window preoperatively, with subsequent postoperative neratinib maintenance; the results of that cohort are forthcoming. We now report the findings of cohorts 3A and 3B, in which neratinib was administered with capecitabine in patients with progressive CNS disease in those without (cohort 3A) and with (cohort 3B) prior lapatinib exposure. TBCRC 022 study cohorts to date have been summarized (Appendix Fig A1, online only). Capecitabine was selected as the therapeutic partner in cohort 3 because of capecitabine’s reported efficacy in CNS and extra-CNS metastatic disease settings, particularly when combined with anti-HER2 therapy, and because of the previously reported activity of neratinib plus capecitabine extracranially.^14-16^ Furthermore, capecitabine has specifically demonstrated CNS penetration of parenchymal tumors in patients receiving capecitabine and lapatinib.^17^

## PATIENTS AND METHODS

### Eligibility

Key eligibility for cohorts 3A and 3B were the following: HER2-positive^18^ breast cancer and measurable CNS metastases (one or more parenchymal brain lesion measuring ≥ 10 mm or more in the longest dimension) with CNS progression after any prior CNS-directed therapy (whole-brain radiotherapy, stereotactic radiosurgery, surgery, CNS-directed systemic therapy, or any combination). Other key inclusion criteria included Eastern Cooperative Oncology Group performance status of 0 to 2, adequate end-organ function, and a cardiac ejection fraction of 50% or more. There was no limit on the number of prior therapy lines, but prior neratinib and capecitabine were not allowed. To explore efficacy by lapatinib exposure, prior lapatinib precluded enrollment in cohort 3A but was a requirement for cohort 3B enrollment. Other key exclusions included escalating steroids over the week before baseline imaging, more than two seizures over the 4 weeks before registration, or any pre-existing chronic, grade 2 diarrhea or greater.

The study was conducted through the TBCRC; all women signed informed consent approved by each institution’s institutional review board. Participating centers included the Dana-Farber (DF)/Harvard Cancer Center (HCC), Baylor College of Medicine, Johns Hopkins University, University of California (San Francisco), University of Michigan, Duke University, University of Pittsburgh, Mayo Clinic, MD Anderson Cancer Center, University of North Carolina, and Georgetown University.

### Treatment Plan

Cohorts 3A and 3B were designed as two separate two-stage, phase II, open-label, single-arm studies. Neratinib was administered at 240 mg orally once per day without breaks, along with capecitabine 750 mg/m^2^ twice per day for 14 days followed by 7 days off. On day 1 of each 21-day cycle, all patients were evaluated with a neurologic examination, including assessment of cranial nerve and motor strength, presence of aphasia or dysphasia, ataxia, somnolence, sensation deficits, and global assessment (worsening, stable, or improved).

Participants were reimaged at baseline and every two cycles with a brain magnetic resonance imaging and computed tomography of the chest, abdomen, and pelvis for the first 18 weeks followed by reimaging every three cycles. Confirmatory scans for response occurred on the same schedule. Patients experiencing non-CNS progression by Response Evaluation Criteria in Solid Tumors (RECIST) 1.1^19,20^ but with stable or responding CNS disease had the option to add trastuzumab. No patients enrolled in the study extension at the time of data cutoff. Mandatory diarrhea prophylaxis included 16 mg of loperamide once per day (4 mg four times per day) during cycle 1 unless constipation or intolerance occurred. All patients received treatment until tumor progression, unacceptable toxicity, severe intercurrent illness, request to come off study, or provider discretion. Correlative studies included plasma cell-free DNA collections at baseline and progression (data forthcoming).

### Definitions of Response

The primary end point was CNS objective response rate (ORR = complete response [CR] + partial response [PR]) for each cohort, according to composite criteria.^15^ An objective composite CNS PR was defined as 50% or greater reduction in the sum of CNS target lesion volumes, without new lesions, non-CNS progression, clearly worsening neurologic status, or increase in corticosteroid dose (for neurologic symptoms). A CR was defined as disappearance of all target lesions plus the other PR criteria. Progressive disease (PD) was defined as a 40% or greater increase in the sum of target lesion volumes compared with the nadir, any new lesion 6 mm or greater, non-CNS progression, worsening neurologic status, or an increase in corticosteroids for neurologic symptoms. Patients who progressed in a non-CNS site first or who died or withdrew from the study for any reason after receiving at least one dose of drug and before a CNS response was determined were considered nonresponders. If neurologic worksheets were missing at reimaging time points, they were not included as part of the composite response assessment. Measurements of CNS lesions were performed centrally by the Harvard Tumor Imaging Metrics Core for all enrolled patients. Non-CNS evaluations were completed by local investigators for outside sites and by Tumor Imaging Metrics Core for DF and HCC sites (all using RECIST 1.1).^19,20^

Secondary objectives included CNS response using the Response Assessment in Neuro-Oncology Brain Metastases (RANO-BM) working group criteria,^21^ with a PR defined as a 30% or greater decrease in the sum of the longest diameters of CNS lesions for 4 weeks or more with no new lesions, stable or improved clinical condition, and stable or decreased corticosteroids, and with PD defined as a 20% increase or greater over the minimum sum of lesions or a new lesion. We also examined progression-free survival (PFS), site of first progression, extracranial responses, overall survival (OS), and toxicity.

### Statistical Analyses

The study had two separate Simon optimal two-stage designs minimizing the expected total accrual under the null for cohorts 3A and 3B, with accrual goals of 35 and 25 patients, respectively. In the first stage of cohort 3A, if five or more of 19 patients had a response, another 16 would enroll. If nine or more of 35 patients achieved a CNS response, the drug combination would be deemed worthy of future study. Under the null hypothesis of 15% ORR, there is a 5% type 1 error, and under the alternative hypothesis of 35%, there is 80% power.

In the first stage of cohort 3B, if two or more of 15 patients had a response, another 10 would enroll. If three or more of 25 had a CNS ORR, then the combination would be deemed promising for lapatinib-treated patients. Under the null hypothesis of 5% ORR, there is a 9% type 1 error, and under the alternative hypothesis of 20%, there is 81% power. These cohorts were designed to assess whether the CNS ORR of the combination was more promising than historical responses for capecitabine alone.^22,23^

In total, 37 patients initiated protocol therapy in cohort 3A (because of robust accrual at the end of the study, with institutional review board permission to overenroll by two patients). For cohort 3B, because of slow accrual in the setting of a changing treatment landscape for metastatic disease (with fewer patients having prior exposure to lapatinib), a decision was made to halt accrual after 12 patients enrolled. All patients were included in analyses. Results are based on the data available as of July 1, 2017. PFS and OS were measured from date of protocol registration and were estimated using Kaplan-Meier curves.

## RESULTS

### Patient characteristics

Between April 2014 and November 2016, 37 patients enrolled in cohort 3A, and between February 2014 and March 2017, 12 patients enrolled in cohort 3B. Patients were enrolled across 10 of the 11 centers. Baseline characteristics are listed in Table 1. Most patients (90%) had an Eastern Cooperative Oncology Group performance status of 0 to 1. The most common extracranial disease sites were bone, lung, and liver. Ninety-two percent of patients in both cohorts had experienced CNS progression after prior CNS-directed therapy. Overall, 32% and 25% of patients in cohorts 3A and 3B had received two or more prior local CNS-directed treatments, respectively.

**TABLE 1.**
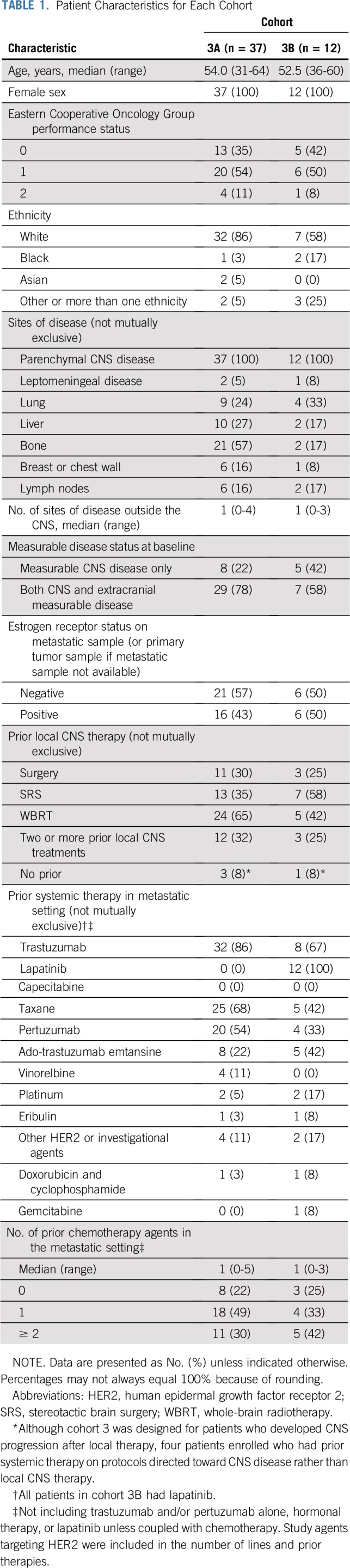
Patient Characteristics for Each Cohort

### Efficacy and Survival

As of July 1, 2017, one patient remained in cohort 3A (cycle 12), and two patients remained in cohort 3B (cycles 9 and 15). In cohort 3A, 21 patients died, two were lost to follow-up, and 14 remained alive. In cohort 3B, six died, one was lost to follow-up, and five remained alive. The median number of cycles initiated was six in cohort 3A (range, one to 30) and five in cohort 3B (range, one to 22); 21 women (57%) in cohort 3A and six (50%) in cohort 3B initiated six or more cycles of therapy.

**FIG 1.**
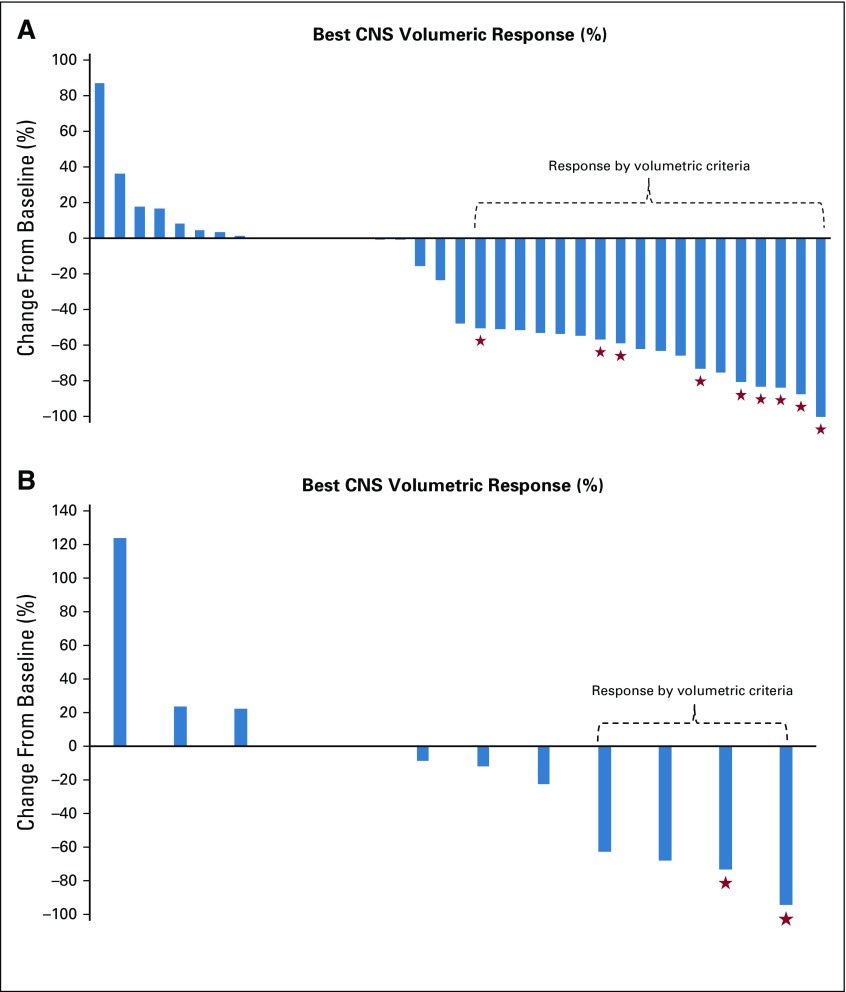
Waterfall plot for best volumetric response in (A) cohort 3A and (B) cohort 3B. Patients who did not make it to their first reimaging were assigned a zero (six patients in cohort 3A: for toxicity [n = 3], MD discretion [n = 1], and clinical CNS progression [n = 2], and two patients in cohort 3B for clinical CNS progression). Stars represent those patients who also had a CNS response by Response Assessment in Neuro-Oncology Brain Metastases (RANO-BM) criteria.

In cohort 3A, nine patients had a composite PR in the first stage (n = 19), and the cohort enrolled to completion plus an additional two patients. Overall, 18 patients had a composite CNS ORR (49% [95% CI, 32% to 66%]); no patients had a CR (Table 2 and Fig 1A). In cohort 3B, four patients had a PR within the first stage before accrual was halted (n = 12; ORR, 33% [95% CI, 10% to 65%]; Table 2 and Fig 1B).

**TABLE 2.**
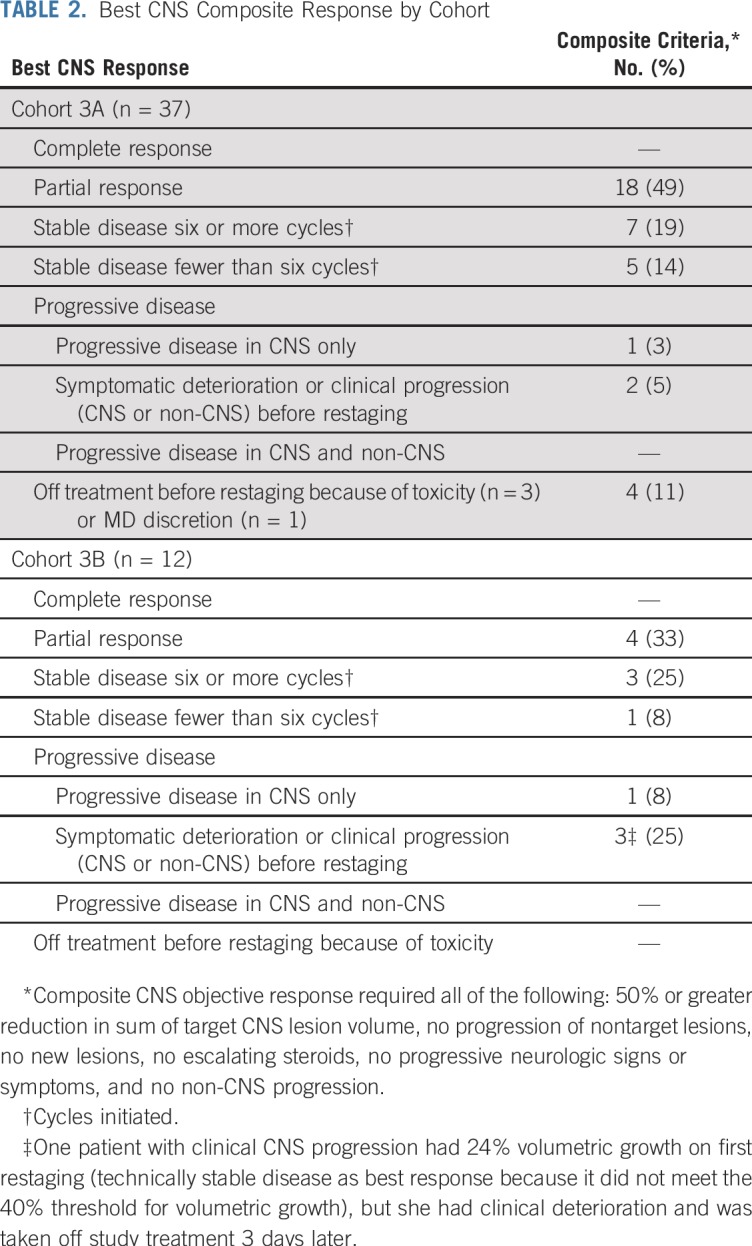
Best CNS Composite Response by Cohort

**FIG 2.**
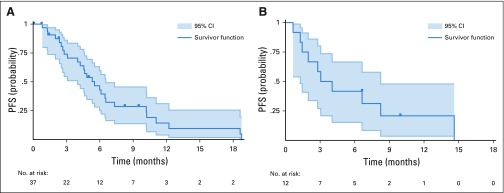
Progression-free survival (PFS) for (A) cohort 3A and (B) cohort 3B.

**FIG A2.**
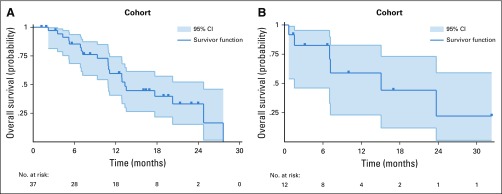
Overall Survival for cohorts (A) 3A and (B) 3B.

In cohort 3A (Fig 2A and Appendix Fig A2A, online only), 11 patients were censored for PFS (six before the first follow-up scan for toxicity, provider discretion, or clinical progression), and median PFS was 5.5 months (range, 0.8 to 18.8 months); 16 patients were censored for OS, and median OS was 13.3 months (range, 2.2 to 27.6 months). In cohort 3B (Fig 2B and Appendix Fig A2B), two participants were censored for PFS, and median PFS was 3.1 months (range, 0.7 to 14.6 months); six were censored for OS, and median OS was 15.1 months (range, 0.8 to 23.7 months). One patient died 20 days after registration (without any follow-up scan), and her PFS was ended by death; all other cases had PFS ended by CNS PD (although four of them at the same time had PD in non-CNS sites).

Among the two patients in cohort 3A and one patient in 3B who presented with leptomeningeal disease as a distinct metastatic site, one developed clinical CNS progression during cycle 1, one had a PR by composite response and continued to receive study treatment of seven cycles, and one had stable disease (SD) as a best response, initiating four cycles of therapy before developing CNS progression. When examining responses by hormone receptor status, 50% of patients with estrogen receptor–positive disease in both cohorts 3A (eight of 16) and 3B (three of six) had a PR by composite criteria. Among those with ER-negative tumors, 43% (10 of 21) had a PR in cohort 3A and 17% (one of six) had a PR in cohort 3B.

**FIG A3.**
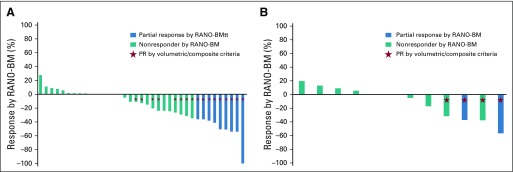
Best Response by Response Assessment in Neuro-Oncology Brain Metastases (RANO-BM) Criteria in (A) Cohorts 3A and (B) 3B Patients who did not make it to their first re-imaging were assigned a zero. Blue bars represent patients who had a CNS response by RANO-BM criteria. Green bars represent patients who did not meet criteria for response by RANO-BM. Red stars represent patients who had a CNS response by composite (volumetric) criteria. On cohort 3A, among the 8 patients who had composite/volumetric responses and not responses by RANO-BM, 6 had stable disease as their best RANO-BM response and 2 had non-sustained partial responses. On Cohort 3B, the 2 patients with composite/volumetric responses met all criteria for RANO-BM response except they were not sustained for at least four weeks.

With regard to CNS response by RANO-BM (Appendix Fig A3, online only), nine patients in cohort 3A had a PR (ORR, 24% [95% CI, 12% to 41%]) and two additional patients had unconfirmed responses that did not persist for 4 weeks or longer. In cohort 3B, two of 12 patients had PR by RANO-BM (ORR, 17% [95% CI, 21% to 48%]). The reasons for discontinuation of study treatment for all study cohorts are listed in Table 3 and were primarily a result of CNS progression; 15% came off study for toxicity, all in cohort 3A.

**TABLE 3.**
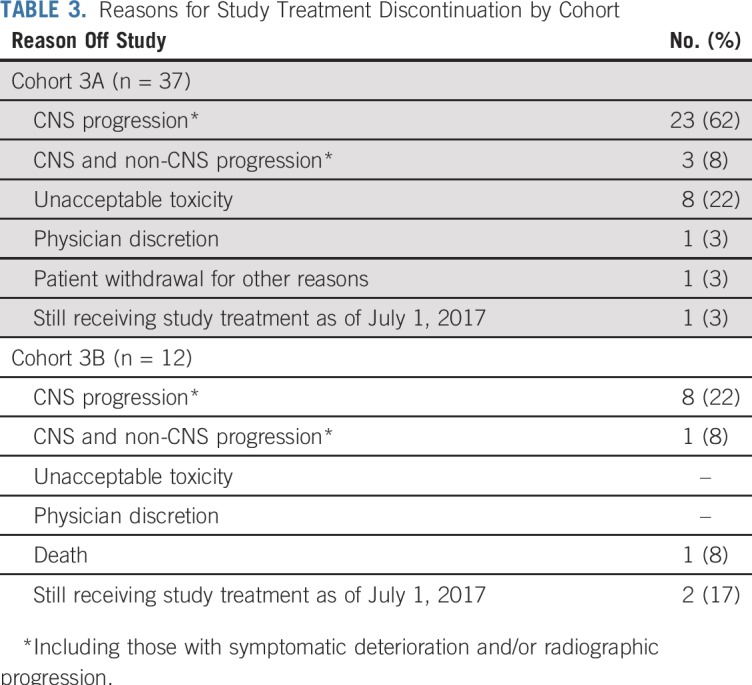
Reasons for Study Treatment Discontinuation by Cohort

With regard to extracranial responses, among the 29 patients in cohort 3A with any measurable extracranial disease at baseline, one had a CR, three had a PR, and 20 had SD per RECIST 1.1; five had baseline scans only because they came off study treatment (extracranial ORR, 14% [95% CI, 4% to 32%]). In cohort 3B, among the seven patients with measurable extracranial disease at baseline, one had a CR, two had a PR, three had SD, and one had no additional scans after baseline (extracranial ORR, 43% [95% CI, 10% to 82%]). As mentioned previously, no patients came off study treatment of extracranial progression alone without simultaneous CNS progression.

### Toxicity

Adverse events by cohort for women in the study deemed possibly, probably, or definitely related to study therapy are listed in Table 4. There were no grade 4 events or greater. The most common adverse event was diarrhea, with 33% having grade 2 and 29% having grade 3 diarrhea in cohorts 3A and 3B combined despite prescribing diarrhea prophylaxis. Grade 2 and 3 (18% and 6%) nausea and/or vomiting (16% and 4%) and fatigue (16% and 10%) were also common, with the remainder of the toxicities occurring less frequently.

**TABLE 4.**
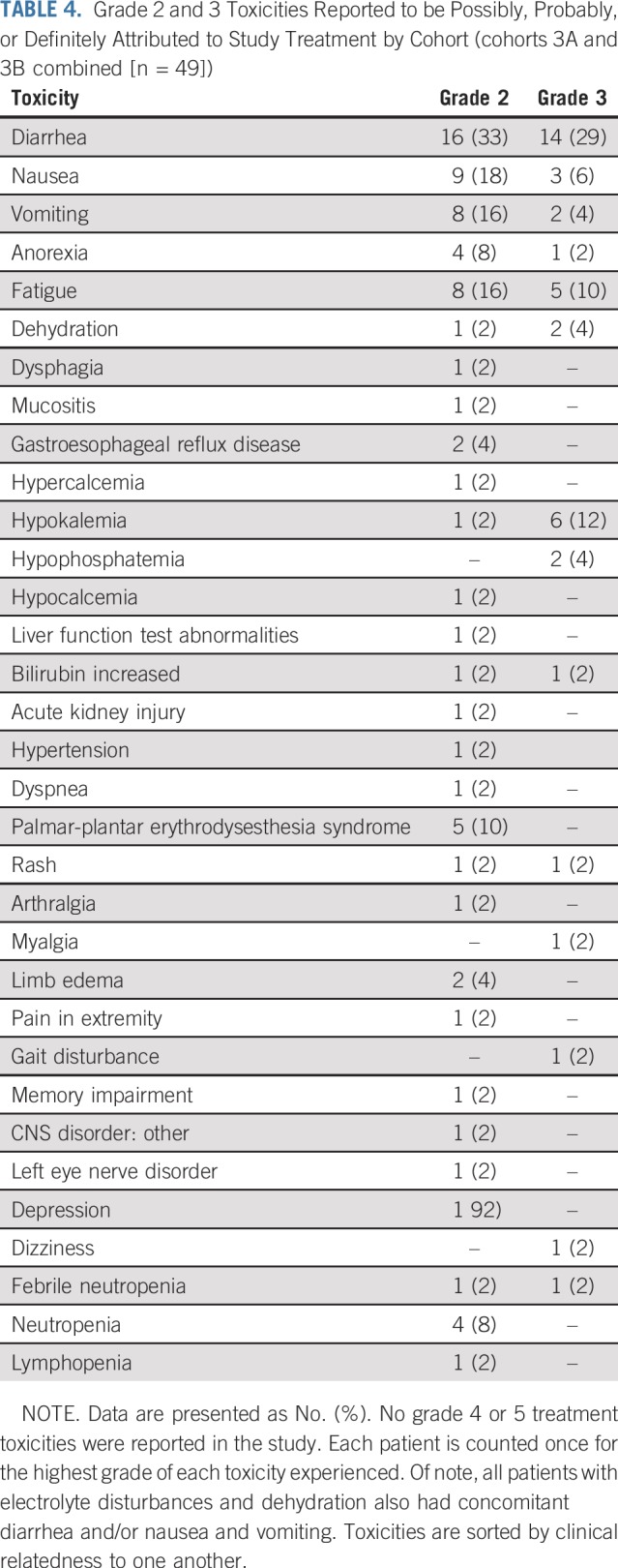
Grade 2 and 3 Toxicities Reported to be Possibly, Probably, or Definitely Attributed to Study Treatment by Cohort (cohorts 3A and 3B combined [n = 49])

Regarding dose modifications, 16 (33%) in cohorts 3A and 3B required neratinib dose reductions. Fifteen of these 16 patients had reductions to 160 mg, with nine patients having this reduction at cycle 2, four having this reduction at cycle 3, one having this reduction at cycle 4, and one initially reducing her dose of neratinib to 200 mg at cycle 2 and then to 160 mg at cycle 3. The 16th patient had a non–protocol-mandated dose reduction of neratinib to 80 mg at cycle 2 because of a strong personal preference. Although dose reductions for capecitabine were also mandated when toxicities were either definitely related to capecitabine (ie, hand-foot rash) or if toxicities possibly could be attributed to either neratinib or capecitabine, we did not systemically collect capecitabine dose reductions.

## DISCUSSION

In this phase II, single-arm, multicohort study, we evaluated the efficacy and toxicity of neratinib and capecitabine in patients with HER2-positive breast cancer brain metastases with and without prior lapatinib exposure. Of note, at baseline, more than 90% of the study population had developed CNS progression despite prior CNS-directed treatment(s), a treatment refractory population. We found that the combination of neratinib and capecitabine was highly active, with nearly one half of participants in cohort 3A experiencing a PR by composite criteria and 57% continuing to receive study therapy for at least six cycles. All patients who stopped treatment of progression experienced CNS progression, even if this occurred concurrently with non-CNS progression, additionally emphasizing the urgent need for developing efficacious treatment options for CNS metastases. We noted numerically similar CNS response rates (50% and 43%) in patients with ER-positive and ER-negative tumors in cohort 3A (lapatinib naïve), and not much difference in the small 3B cohort (three of six *v* one of six). Given the small numbers of patients, we had too little power to confirm or refute ER status findings from Neratinib after trastuzumab-based adjuvant therapy in HER2-positive breast cancer.^24^ Similarly, the number of patients with leptomeningeal disease was too small to draw firm conclusions. Neratinib and capecitabine were active in those with prior lapatinib exposure, with a composite CNS ORR of 33%, although the confidence interval is wide because of early closure of this cohort. Overall, our observed CNS response rate of 49% exceeds the point estimates for either neratinib monotherapy (8%)^11^ or capecitabine and temozolomide (18%).^22^

After the start of this study, RANO-BM emerged as a standardized approach to assessment for CNS response.^21^ Although volumetric response continues to provide important information, it is not often available in real time at many centers and adds cost and complexity to radiologic review. In our study, CNS RANO-BM responses in cohorts 3A and 3B were lower (24% and 17%), partly because of the lack of verification of response duration of 4 weeks or more in four patients (two in cohort 3A and two in 3B) and partly because the RANO-BM criteria for PR are stricter than volumetric criteria (six patients in cohort 3A). In general, if a tumor is a perfect sphere, a 30% reduction in diameter will correspond to an approximate 65% volumetric reduction. Thus, a 50% reduction in volume does not consistently predict for a response by diameter.^21^

The combination of neratinib and capecitabine has been reported previously to result in extracranial ORRs of 64% and 57%, respectively, in lapatinib-naïve and lapatinib-pretreated patients without brain metastases.^16^ The soon-to-be-reported Study of Neratinib Plus Capecitabine Versus Lapatinib Plus Capecitabine in Patients With HER2+ Metastatic Breast Cancer Who Have Received Two or More Prior HER2 Directed Regimens in the Metastatic Setting (NALA) study (ClinicalTrials.gov identifier: NCT01808573) is comparing the combination of neratinib and capecitabine with lapatinib and capecitabine and will provide data regarding the relative efficacy of these combinations. Unfortunately, the study excluded patients with symptomatic or unstable brain metastases; hence, even when the study is reported, we will not have direct evidence comparing the regimens in patients with CNS disease. Nevertheless, our results compare favorably to those reported previously with lapatinib and capecitabine. Using identical CNS composite criteria for response in a similar patient population, prospective phase II trials of lapatinib and capecitabine have reported response rates of 20% to 38%, with median PFS of 3.6 months.^15,25^ Our results also compare favorably to the investigator-choice control arm of the LUX Breast-3 trial, which reported a CNS ORR of 14% (using RECIST 1.1) and a PFS of 18.4 weeks in patients with HER2-positive brain metastases.^26^ Although the CNS ORR in our study was lower than that reported in the Lapatinib plus capecitabine in patients with previously untreated brain metastases from HER2-positive metastatic breast cancer (LANDSCAPE) trial with lapatinib and capecitabine,^14^ our study included patients who had CNS progression after prior CNS-directed therapy. Evaluating the efficacy of neratinib-based combinations in the up-front CNS disease setting is of interest and will be studied in future cohorts.

With regard to toxicity, nearly 30% of patients receiving neratinib and capecitabine experienced grade 3 diarrhea despite loperamide prophylaxis, and eight patients (22%) came off treatment of toxicity in cohort 3A, perhaps limiting PFS evaluations. However, we did not capture loperamide adherence or use of other antidiarrheal agents, and it is possible that patients did not adhere to their prophylaxis. It is possible that selection bias affected toxicity events, because no patients in cohort 3B stopped treatment of toxicity. In future planned TBCRC 022 cohorts, we will standardize the way diarrhea events and the use of mandated and as-needed antidiarrheals are captured. Furthermore, new regimens may be more effective than loperamide alone and should be explored to minimize toxicity. For example, in the Effects of adding budesonide or colestipol to loperamide prophylaxis on neratinib-associated diarrhea in patients with HER2+ early-stage breast cancer (CONTROL) study,^27^ the lowest incidence of grade 3 diarrhea (10.8%) occurred in patients receiving loperamide in combination with colestipol.

We recognize several study limitations. Our study did not include a comparison arm, and study participants had a varied set of past CNS-directed treatments and systemic therapy exposures, although this likely represents the diversity of patients we see in practice. Our study population was generally heavily pretreated, with a high burden of systemic disease in both cohorts (in addition to CNS disease). Also, because of small sample sizes, a detailed examination of factors associated with response was not possible. Last, we did not systematically collect dose modifications of capecitabine, limiting our ability to quantify the dose intensity for this drug.

Beyond these specific trial results, the steady accrual of this trial highlights the unmet medical need for new therapies for brain metastases and the feasibility of conducting trials in this patient population. In total, we have enrolled 96 patients in all TBCRC 022 cohorts to date. Our results provide additional support for the efficacy of HER2-directed systemic therapy for the treatment of breast cancer brain metastases. Future studies could examine local therapy versus systemic therapy in CNS disease and additionally explore the role of other neratinib-based combination regimens. Indeed, we will be testing the combination of neratinib with ado-trastuzumab emtansine in the hope of enhancing efficacy and perhaps mitigating diarrhea-related toxicity. Additional study of potentially active CNS agents is crucial to improve the quality and duration of life for patients with progressive metastatic breast cancer to the brain.

In summary, we report substantial CNS activity and some durable responses to the combination of neratinib and capecitabine in patients with progressive HER2-positive breast cancer brain metastases. Although toxicity remains a concern, our results have led to the inclusion of neratinib and capecitabine as a recommended treatment option for HER2-positive CNS disease in the National Comprehensive Cancer Network’s CNS Cancers guidelines.^28^ We await the data from the NALA study to inform our understanding of the relative merits of neratinib- versus lapatinib-based regimens for the treatment of patients with progressive extracranial disease.

## Data Availability

The following represents disclosure information provided by authors of this manuscript. All relationships are considered compensated. Relationships are self-held unless noted. I = Immediate Family Member, Inst = My Institution. Relationships may not relate to the subject matter of this manuscript. For more information about ASCO's conflict of interest policy, please refer to www.asco.org/rwc or ascopubs.org/jco/site/ifc. **Research Funding:** Puma Biotechnology (Inst), Eisai (Inst) **Consulting or Advisory Role:** Novartis, Merrimack, Genentech/Roche, Eli Lilly, Nektar, Seattle Genetics, Puma Biotechnology **Research Funding:** Novartis (Inst), Puma Biotechnology (Inst), Merrimack (Inst), Eli Lilly (Inst), Merck (Inst), Nektar (Inst), Tesaro (Inst), Seattle Genetics, G1-Therapeutics **Patents, Royalties, Other Intellectual Property:** Up to Date.com, Jones and Bartlell **Stock and Other Ownership Interests:** Merrimack (I) **Honoraria:** Agendia NV, Genentech (I), Pfizer (I) **Speakers' Bureau:** Genentech (I), Agendia NV, Pfizer (I) **Research Funding:** Genentech (Inst), Galena Biopharma (Inst), Celldex (Inst), Puma Biotechnology (Inst), Eli Lilly (Inst) **Patents, Royalties, Other Intellectual Property:** Multiple patents related to immunoliposomal drugs being studied and potentially brought to market by Merrimack (I) **Stock and Other Ownership Interests:** US WorldMeds **Research Funding:** Genentech/Roche (Inst), Puma Biotechnology (Inst), Novartis (Inst), Merrimack (Inst), Clovis Oncology (Inst), Merck (Inst), Macrogenics (Inst) **Travel, Accommodations, Expenses:** Syndax Pharmaceuticals **Consulting or Advisory Role:** MOTUS (I)m Remedy Partners (I), Dark Canyon (I) **Research Funding:** BayerAG (Inst) **Patents, Royalties, Other Intellectual Property:** UpToDate **Employment:** Eli Lilly **Consulting or Advisory Role:** Roche **Research Funding:** Genentech (Inst), Pfizer (Inst), Novartis (Inst) **Travel, Accommodations, Expenses:** Celgene, Novartis, Pfizer, Roche, Sandoz, Genentech, Amgen, Eli Lilly, Advaxis, Coherus Biosciences, Eisai, Macrogenics, Merck, Pierian Biosciences, Puma Biotechnology **Consulting or Advisory Role:** Abbvie, MedImmune, Celldex, Puma Biotechnology, Pfizer, AstraZeneca, Eisai, NanoString Technologies **Research Funding:** Abbvie (Inst), Pfizer (Inst), Eli Lilly (Inst), Novartis (Inst), Incyte (Inst), Covance/Bayer (Inst), AstraZeneca (Inst), Genentech (Inst), Medivation (Inst) **Honoraria:** Eisai **Stock and Other Ownership Interests:** Abbvie (I), Abbott Laboratories (I) **Honoraria:** AsiM CME **Research Funding:** Pfizer (Inst), Galena Biopharma (Inst), Syndax Pharmaceuticals (Inst), Novartis (Inst), AstraZeneca (Inst) **Honoraria:** Celgene, Roche, Novartis **Consulting or Advisory Role:** Immunomedics **Speakers' Bureau:** Celgene, Roche, Novartis **Research Funding:** Nektar **Travel, Accommodations, Expenses:** Roche, Novartis **Consulting or Advisory Role:** Puma Biotechnology **Honoraria:** Genomic Health **Consulting or Advisory Role:** Novartis, Immunomedics **Consulting or Advisory Role:** AstraZeneca **Research Funding:** AstraZeneca **Leadership:** Immunonet BioSciences **Stock and Other Ownership Interests:** Immunonet BioSciences **Consulting or Advisory Role:** Personalized Cancer Therapy, OncoPlex Diagnostics, Immunonet BioSciences, Pfizer, HERON, Puma Biotechnology **Speakers' Bureau:** Genentech/Roche **Research Funding:** Genentech/Roche (Inst), Genentech/Roche (Inst), Fabre-Kramer (Inst), Advanced Cancer Therapeutics (Inst), Caris Centers of Excellence (Inst), Caris Centers of Excellence (Inst), Pfizer (Inst), Pieris Pharmaceuticals (Inst), Cascadian Therapeutics (Inst), Pfizer (Inst) **Patents, Royalties, Other Intellectual Property:** United States Patent no. 8,486,413, United States Patent no. 8,501,417, United States Patent no. 9,023,362, United States Patent no. 9,745,377 **Employment:** The Emmes Corporation **Consulting or Advisory Role:** Abbvie (I) **Consulting or Advisory Role:** Genentech, Novartis, Macrogenics, Daiichi Sankyo **Research Funding:** Pfizer (Inst) **Employment:** AMAG Pharmaceuticals (I) **Leadership:** AMAG Pharmaceuticals (I) **Stock and Other Ownership Interests:** AMAG Pharmaceuticals (I) **Honoraria:** Genentech/Roche **Consulting or Advisory Role:** Genentech/Roche, Daiichi Sankyo, Context Therapeutics, Macrogenics, Taiho Pharmaceutical **Research Funding:** Genentech/Roche (Inst), Seattle Genetics (Inst), Pfizer (Inst), Daiichi Sankyo (Inst) **Research Funding:** Myriad Genetics (Inst), Pfizer (Inst), Biomarin (Inst), Celldex (Inst) **Patents, Royalties, Other Intellectual Property:** Antonio Wolff has been named as inventor on one or more issued patents or pending patent applications relating to methylation in breast cancer, and has assigned his rights to JHU, and participates in a royalty sharing agreement with JHU **Honoraria:** Genentech/Roche, Tesaro, Eli Lilly **Consulting or Advisory Role:** Leap Therapeutics, InfiniteMD **Research Funding:** Genentech (Inst) **Consulting or Advisory Role:** Genentech/Roche, Seattle Genetics, Puma Biotechnology, Shionogi, Novartis **Research Funding:** Genentech, Pfizer, Novartis, Array BioPharma **Patents, Royalties, Other Intellectual Property:** Royalties for chapter in Up-to-Date regarding management of breast cancer brain metastases No other potential conflicts of interest were reported.
